# Hold your horSSEs: controlling structure-selective endonucleases MUS81 and Yen1/GEN1

**DOI:** 10.3389/fgene.2015.00253

**Published:** 2015-07-30

**Authors:** Miguel G. Blanco, Joao Matos

**Affiliations:** ^1^Department of Biochemistry and Molecular Biology, Center for Research in Molecular Medicine and Chronic Diseases, University of Santiago de Compostela, Santiago de Compostela, Spain; ^2^Institute of Biochemistry, Swiss Federal Institute of Technology in Zürich, Zürich, Switzerland

**Keywords:** nuclease, DNA repair, recombination, replication, Holliday junction, Cdk, Cdc5/PLK1, Cdc14

## Abstract

Repair of DNA lesions through homologous recombination promotes the establishment of stable chromosomal interactions. Multiple helicases, topoisomerases and structure-selective endonucleases (SSEs) act upon recombining joint molecules (JMs) to disengage chromosomal connections and safeguard chromosome segregation. Recent studies on two conserved SSEs – MUS81 and Yen1/GEN1– uncovered multiple layers of regulation that operate to carefully tailor JM-processing according to specific cellular needs. Temporal restriction of SSE function imposes a hierarchy in pathway usage that ensures efficient JM-processing while minimizing reciprocal exchanges between the recombining DNAs. Whereas a conserved strategy of fine-tuning SSE functions exists in different model systems, the precise molecular mechanisms to implement it appear to be significantly different. Here, we summarize the current knowledge on the cellular switches that are in place to control MUS81 and Yen1/GEN1 functions.

## Establishment and Safe Removal of DNA Joint Molecules During Recombinational DNA Repair

In all organisms, the preservation of hereditary information relies on repair mechanisms that counteract the lesions constantly inflicted on their DNA. Cells have matched the diversity and complexity of these injuries with a staggering assortment of DNA repair pathways specialized in specific types of damage. While insults like chemical modifications of the nucleotide bases and single-strand breaks are some of the most abundant (Lindahl and Barnes, 2000), DNA double-strand breaks (DSBs) pose a higher risk for the cell, as failure to repair them may lead to the loss of whole chromosomal arms.

**FIGURE 1:**
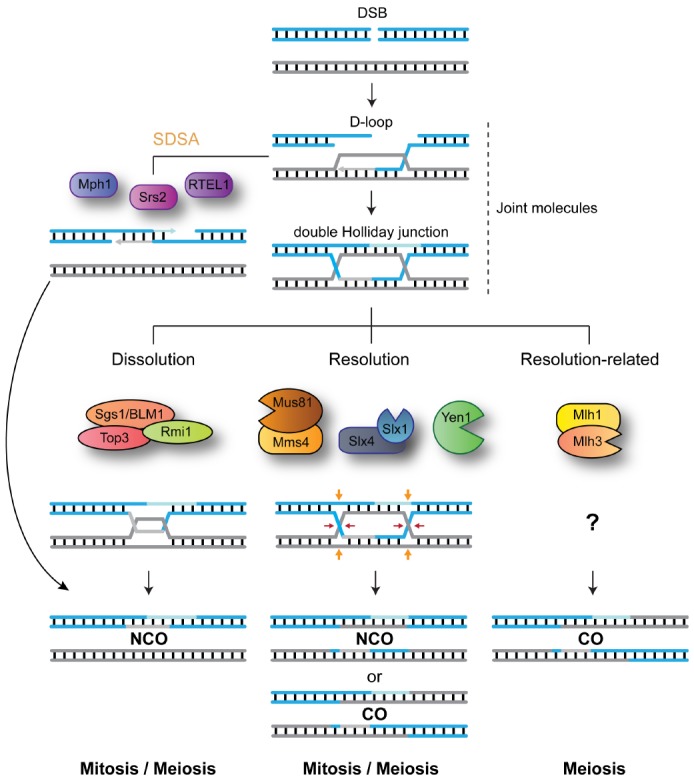
**DNA-centric model for JM metabolism during mitotic and meiotic double-strand break (DSB) repair.** After DNA-end resection, strand invasion leads to the formation of joint molecules (JMs) containing displacement loops (D-loops). Unwinding of the invading strand, mediated by Srs2, Mph1 or RTEL1, mediates synthesis-dependent strand annealing (SDSA) and the formation of NCO recombinants. Alternatively, capture of the second broken DNA end, by the D-loop structure, precedes double Holliday junction formation. The STR complex dissolves double Holliday junctions to generate NCO recombinants. Mus81-Mms4/EME1, Slx1-Slx4, and Yen1/GEN1 resolve HJs by endonucleolytic cleavage to generate COs and NCOs. Mlh1-Mlh3 process HJs to generate exclusively COs. For simplicity, the roles for Sgs1 helicase in processing early JMs and in promoting meiotic CO formation are not depicted.

Homologous recombination (HR) is an evolutionarily conserved DSB repair pathway that resorts to an intact DNA molecule with an identical (or nearly identical) sequence, such as the sister chromatid or the homologous chromosome, to restore the integrity of the broken strands. For this purpose, the HR machinery drives the damaged DNA duplex through a series of molecular exercises that include DNA end-resection, homology search, strand invasion and DNA synthesis to retrieve the missing information (Heyer et al., 2010; Symington and Gautier, 2011). One central feature of HR is that pairing and strand exchange reactions lead to the formation of increasingly stable recombination intermediates. At the chromosomal level, these structures translate into inter-sister (or inter-homolog) DNA joint molecules (JMs) that must be disconnected prior to cell division. To solve this problem, cells frequently employ anti-recombinogenic helicases that dislodge the invading DNA strand on displacement loop structures (D-loops; Figure 1). However, long-lived D-loops may occasionally capture the second broken end, which then primes DNA synthesis using the displaced strand as a template. Sealing of the nicks at the end of the newly synthesized strands leads to the establishment of fully ligated four-way junctions, termed Holliday junctions (HJs; Liu and West, 2004; Holliday, 2007). Due to the covalent nature of the link that is formed as they mature, HJs are arguably the most dangerous of all recombination intermediates that contribute to the linkage of two DNA duplexes. It is important to point out that such potentially dangerous intermediates appear not only as a consequence of DSB repair, but also during DNA replication (Giannattasio et al., 2014), since HR is also involved in both replication fork reactivation and post-replicative ssDNA gap-filling (Branzei and Foiani, 2010; Rass, 2013).

Despite the risks of interlocking the two recombining chromosomes, the formation of JMs is crucial for HR repair both during mitosis and meiosis. Maturation of early recombination intermediates into HJs within meiotic JMs precedes the formation of crossovers (COs), repair products characterized by the physical exchange of the DNA duplexes flanking the branch point (Schwacha and Kleckner, 1995; Allers and Lichten, 2001; Petronczki et al., 2003). Importantly, COs not only result in the reassortment of genetic information between maternal and paternal genomes, but are also required for the correct bipolar segregation of homologs during the first meiotic division (Schwacha and Kleckner, 1995; Allers and Lichten, 2001; Petronczki et al., 2003). Since inter-homolog exchanges can lead to loss-of-heterozygosity (LOH), mitotic cells disengage most JMs at an early stage to prevent CO formation (Ira et al., 2003; Bzymek et al., 2010). In addition, during the mitotic cell cycle, removal of late JMs that contain HJs is biased toward pathways that promote formation on non-crossover recombinants (NCOs; Dayani et al., 2011).

So how do cells modify JM-processing according to the specialized cellular needs of mitosis and meiosis? In order to efficiently disengage recombination intermediates, while having flexibility toward the choice of recombination outcome (CO vs. NCO), cells have evolved a blend of DNA-processing enzymes with specialized abilities (Figure 1). Helicases, such as Srs2, Mph1/FANCM, or RTEL1, are capable of unwinding early recombination intermediates like D-loop structures to generate exclusively NCO recombinants (Ira et al., 2003; Barber et al., 2008; Prakash et al., 2009). Structure-selective endonucleases (SSEs), such as MUS81-EME1 (Mus81-Mms4 in *Saccharomyces cerevisiae*; Mus81-Eme1 in *Schizosaccharomyces pombe*; hereinafter, we will use the term MUS81* to refer to all these orthologs collectively), SLX1-SLX4 (Slx1-Slx4 in *S. cerevisiae* and *S. pombe*) and GEN1 (Yen1 in *S. cerevisiae*; absent in *S. pombe*), can cleave late recombination intermediates, containing HJs or HJ precursors, to generate a mixture of COs and NCOs (Boddy et al., 2000, 2001; Kaliraman et al., 2001; Fricke and Brill, 2003; Ip et al., 2008; Andersen et al., 2009; Fekairi et al., 2009; Munoz et al., 2009; Svendsen et al., 2009; Schwartz and Heyer, 2011). The STR complex (BTR in humans), composed of the RecQ helicase Sgs1 (BLM), the topoisomerase Top3 (TOP3α), and Rmi1 (RMI1/2), promotes the convergent branch-migration and decatenation of double HJs to generate NCOs (Gangloff et al., 1994; Fabre et al., 2002; Wu and Hickson, 2003). Finally, the Mlh1-Mlh3 nuclease mediates HJ processing to generate exclusively COs through a mechanism that remains elusive (Zakharyevich et al., 2012; Ranjha et al., 2014; Rogacheva et al., 2014; Figure 1).

Despite the identification of specialized pathways in JM-processing, most (perhaps all) JM-processing enzymes are expressed and function during mitosis and meiosis. Therefore, one key question that arises and remains largely unanswered is: how do cells tailor pathway usage to satisfy their specialized needs? Recent studies focusing on MUS81* and Yen1/GEN1, two SSEs with important roles in mitotic and meiotic DNA repair, have started to unveil the subtle manipulations that cells utilize to tame their potentially deleterious activities, blocking or unleashing them according to their particular requirements. In the next sections, we will attempt to summarize the current knowledge on the mechanisms employed to control SSE function.

## Regulation of MUS81* and Yen1/GEN1 Structure-Selective Nucleases

“Edged tools are dangerous things to handle, and not infrequently do much hurt”

– Agnes Repplier (1855–1950)

### The MUS81* Nucleases

**FIGURE 2:**
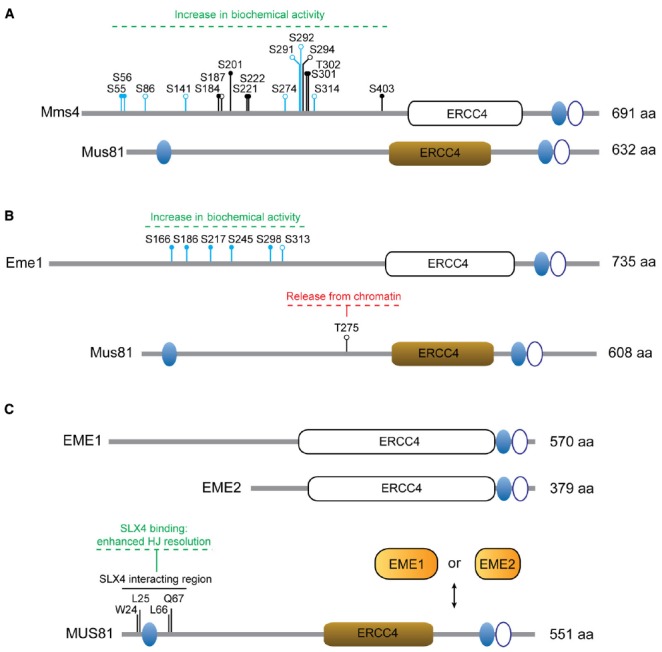
**Regulation of MUS81 complexes. (A)** Mus81 and Mms4 from *S. cerevisiae*. The residues modified in the different Mms4 mutants are depicted: mms4-7A (S56A, S184A, S201A, S222A, S294A, T302A, S403A); mms4-np (S55A, S56A, S184A, S201A, S221A, S222A, S301A, T302A, S403A); mms4-14A (S55, S56, S86, S141, S184, S187, S201, S274, S291, S292, S301, T302, S314, S403). **(B)** Mus81 and Eme1 from *S. pombe*. The residues modified in Eme1^6SA^ are shown (S166A, S186A, S217A, S245A, S298A, S313A) and include those in Eme1^4SA^ (S166A, S186A, S217A, S245A). **(C)** Human MUS81, EME1 and EME2. Non-functional ERCC4 motifs are depicted as white boxes. Ovals represent functional (filled) or non-functional (open) HhH motifs. Relevant amino acid residues and the effects of particular modifications are indicated. Close circles denote consensus sites for Cdk phosphorylation or Cdc5 binding sites. Open circles denote phosphorylation sites. Residues in blue have been identified as phosphosites *in vivo* by mass-spectrometry.

MUS81* belongs to the XPF/Rad2 family of nucleases, whose structural and functional features have been superbly reviewed elsewhere (Ciccia et al., 2008). Therefore, we will only briefly highlight some of its most relevant characteristics for our topic. Like all the other members of the family, MUS81* exists as a heterodimeric protein complex and harbors the distinctive ERCC4 nuclease domain, in addition to helix-hairpin-helix (HhH) motifs in both the N-terminal and C-terminal regions (Figure 2). Its partner proteins (Mms4 in budding yeast, Eme1 in fission yeast, EME1 and EME2 in human cells) have a similar domain organization, with exception of the absence of the N-terminal HhH motif. Despite being indispensable for MUS81* stability and the nuclease activity of the complex, Mms4, Eme1, EME1, and EME2 are regarded as non-catalytic subunits because they contain mutations in key residues of the ERCC4 domain.

In *S. cerevisiae*, *mms4* mutants were initially described by their increased sensitivity to the alkylating agent methyl methanesulfonate (Xiao et al., 1998) and Mus81 was isolated as a specific interactor of Rad54 in a yeast two-hybrid screen (Interthal and Heyer, 2000). Both genes were also recovered in a synthetic lethality screen of *sgs1*Δ mutants (Mullen et al., 2001). In *S. pombe*, Mus81 was identified through a yeast two-hybrid approach as an interactor of the checkpoint kinase Cds1 and found to exist in a complex with Eme1 (Boddy et al., 2000, 2001). Due to its high conservation, bioinformatic analyses succeeded in recognizing Mus81 orthologs in other organisms, including humans (Chen et al., 2001). The strong mitotic and meiotic phenotypes of *mus81*Δ and *eme1*Δ/*mms4*Δ mutants, including impaired DNA-damage repair, reduced spore viability and crossover formation led to the proposal that the MUS81* nucleases were the eukaryotic HJ resolvases (Boddy et al., 2000, 2001; de los Santos et al., 2001, 2003; Mullen et al., 2001). However, this view was controversial since the biochemical properties of these nucleases suggested a different resolution mechanism from the well-established bacterial resolvase RuvC, a homodimeric protein that introduces two symmetrical nicks in strands of like polarity across one axis of the HJ, yielding nicked DNA duplexes that can be ligated without the need of further processing (West, 1997; Haber and Heyer, 2001; Heyer et al., 2003; Heyer, 2004; Hollingsworth and Brill, 2004). MUS81* complexes from different organisms can cleave a number of different branched structures efficiently, including 3′-flaps, D-loops, model replication forks and nicked HJs, while intact HJs are generally poor substrates for this nuclease (Boddy et al., 2001; Kaliraman et al., 2001; Constantinou et al., 2002; Doe et al., 2002; Ciccia et al., 2003; Gaillard et al., 2003; Ogrunc and Sancar, 2003; Osman et al., 2003; Fricke et al., 2005; Gaskell et al., 2007; Ehmsen and Heyer, 2008; Schwartz et al., 2012; Pepe and West, 2014b). Given the broad spectrum of branched structures that the MUS81 complexes can target, it was soon proposed that without strict regulation its activity might not be beneficial and give rise to potentially deleterious events (Kai et al., 2005). Interestingly, recent work from different model organisms indicates that the biological roles of the MUS81 nucleases are carefully modulated by post-translational modifications. This allows cells to tailor MUS81 function according to specific cellular needs, such as boosting its ability to process JMs that have persisted until the mitotic stage, while avoiding the unscheduled processing of other physiologically important branched DNA structures.

#### *S. cerevisiae* Mus81-Mms4

In budding yeast, Mus81 is associated to the non-catalytic subunit Mms4, with their main interaction domain residing in the C-terminal region of both proteins (Fu and Xiao, 2003). It has been found that the biochemical activity of Mus81-Mms4 fluctuates throughout the cell cycle both in meiotic and mitotic cells, from a minimum in G1/S to a maximum at G2/M (Matos et al., 2011; Matos and West, 2014). As cells approach M-phase, Mms4 is increasingly phosphorylated, with a concomitant boost in the catalytic activity of the complex (Matos et al., 2011, 2013; Gallo-Fernandez et al., 2012; Szakal and Branzei, 2013). When the hyperphosphorylated Mus81-Mms4 from cells in G2/M is dephosphorylated *in vitro*, its nuclease activity decreases to a basal level, indicating that biochemical hyperactivation is a direct consequence of phosphorylation (Matos et al., 2011).

Two cell-cycle kinases have been implicated in both events: the Polo kinase Cdc5 and M-phase Cdc28/Cdk (Matos et al., 2011, 2013; Gallo-Fernandez et al., 2012; Szakal and Branzei, 2013). Whereas both kinase activities are required for Mus81-Mms4 activation at the G2/M transition, Cdc5 activity seems to be especially relevant as Cdc5 overexpression is sufficient to drive phospho-activation outside M-phase (Matos et al., 2011, 2013). Furthermore, Cdc5 kinase is sufficient to hyperactivate Mus81-Mms4 *in vitro* (Schwartz et al., 2012). However, the precise contributions of each of these kinases to Mus81-Mms4 regulation *in vivo* remain to be determined: is Cdk-mediated phosphorylation important only in priming Mms4 for Cdc5 binding, or does it also have a more direct role in the regulation of nuclease activity?

The generation of Mms4 mutants in which Cdk/Cdc5-dependent modification is impaired has helped us understand the biological relevance of Mus81-Mms4 phosphorylation (Figure 2A). In this sense, the *mms4-7A* (Szakal and Branzei, 2013) and *mms4-np* (Gallo-Fernandez et al., 2012) alleles were created to encode substitutions of serines or threonines at predicted Cdk (S/T-P) or Cdc5-docking (S-pS/pT-P) consensus sites in the sequence of *MMS4*. An additional mutant, *mms4-14A*, was engineered to prevent phosphorylation in both predicted and *in vivo*-validated phosphoresidues found in Mms4 from nocodazole-arrested cells (Matos et al., 2011). As expected, all three mutants are largely resistant to mitosis-specific phosphorylation (Matos et al., 2011, 2013; Gallo-Fernandez et al., 2012; Szakal and Branzei, 2013). To assess if modification of Mms4 could influence the catalytic properties of Mus81-Mms4, the nuclease activities of Mus81-mms4-np and Mus81-mms4-14A were measured in immunoprecipitates from synchronous cells at different stages of the cell cycle. Both mutants displayed impaired nuclease activation at the G2/M transition, consistent with the idea that Cdc28/Cdk and Cdc5-mediated Mms4 modification is required for Mus81 hyperactivation (Matos et al., 2011; Gallo-Fernandez et al., 2012). Although it has not been formally tested, it is expectable that mms4-7A will manifest similar properties.

The phenotypic analysis of *mms4-7A* and *mms4-14A* mutant strains revealed a strong sensitivity to DNA-damaging agents and a severe synthetic growth defect when combined with *sgs1*Δ (Matos et al., 2011, 2013; Szakal and Branzei, 2013). Both phenotypes are also shared with *cdc5-2* mutants, which are unable to phosphorylate and activate Mus81-Mms4 during mitosis (Matos et al., 2013). This is in contrast to *mms4-np* mutants, which show considerably milder phenotypes and only display increased sensitivity to genotoxic agents in the absence of *sgs1*Δ (Gallo-Fernandez et al., 2012). It is yet unclear why the *mms4-np*, the mutant with an intermediate number of alanine substitutions -nine-, has a milder phenotype than both the *mms4-7A* and *mms4-14A* alleles, but it has been proposed that differences in the genetic backgrounds employed by each group may account for this fact (Szakal and Branzei, 2013). Altogether, these results indicate that hyperactivation of the Mus81-Mms4 nuclease at G2/M is important for the elimination of those recombination intermediates that escape the action of Sgs1.

From a mechanistic point of view, how the phosphorylation of the nuclease drives its hyperactivation is not yet understood. It has been proposed that Mus81-Mms4 may exist in dimeric as well as tetrameric states in solution (Gaskell et al., 2007). This hypothesis would provide an intuitive and elegant system for its hyperactivation, particularly in HJ processing, which requires a double incision for resolution of the X-shaped structure. However, *in vitro*-phosphorylation experiments with purified Mus81-Mms4 and Cdc5, followed by size-exclusion chromatography, have ruled out that the phosphorylation-dependent hyperactivation is a result of multimerization of the nuclease (Schwartz et al., 2012). An alternative possibility is that phosphorylation may lead to changes in the stability of the complex. This seems unlikely, though, as most of the mapped and predicted phosphorylation sites lie outside the interaction domain between Mms4 and Mus81, and mms4-14A seems to associate normally with Mus81 (Matos et al., 2011). Finally, phosphorylation events could trigger a structural change that favors binding and/or turnover of the enzyme-substrate complex. Interestingly, two phosphomimetic mutants have been proposed to represent constitutively active versions of the Mus81-Mms4 nuclease (*mms4-56E* and *mms4-56E*, *184D*), as their expression promotes increased CO formation and reduced accumulation of X-shaped molecules in *sgs1*Δ mutants (Szakal and Branzei, 2013). In the future, analysis of the biochemical properties of such mutants may contribute to our understanding of the mechanism of Mus81-Mms4 nuclease activation.

At the level of protein–protein interactions, it has been reported that the binding of the N-terminal region of Mus81 and the C-terminal region of Rad27/FEN1 results in their mutual enzymatic stimulation (Kang et al., 2010; Thu et al., 2015). In addition, while the human orthologs of the Mus81-Mms4 and Slx1-Slx4 can physically interact (see Human MUS81-EME1/EME2), the initial results in budding yeast showed that these complexes could not associate nor stimulate each other *in vitro*, at least for the cleavage of different HJ substrates and model replication forks (Schwartz et al., 2012). However, more recent work has shown that Slx1-Slx4 can stimulate Mus81-Mms4 activity on 3′-flap structures (Thu et al., 2015). Furthermore, phosphorylation of the Mus81-Mms4 by Cdc5 leads to its association with the scaffold protein Dpb11 at G2/M, which can also interact with Slx4 (Gritenaite et al., 2014). While it has not been demonstrated that the formation of this Mus81-Mms4-Dpb11-Slx4 complex alters the biochemical properties of Mus81-Mms4, it may provide a system for substrate targeting, rendering Mus81 a more efficient nuclease *in vivo*.

Altogether, the emerging picture is that cell cycle stage-specific phosphorylation events are likely to modulate Mus81-Mms4 function through several complementary mechanisms: (1) direct enhancement of nuclease activity; (2) regulation of nuclease activity through stimulatory protein–protein interactions; (3) regulation of protein–protein interactions that facilitate recruitment to cognate substrates.

#### *S. pombe* Mus81-Eme1

The Mus81-Eme1 complex from fission yeast was the first eukaryotic nuclease to be considered a nuclear HJ resolvase (Boddy et al., 2001). Interestingly, the initial description of Mus81 as an interactor of the checkpoint kinase Cds1 (Rad53, CHK2) also revealed that Mus81 is indeed modified by Cds1 after hydroxyurea (HU) treatment (Boddy et al., 2000). The phosphorylation of Mus81 at T275 (T239 in the original manuscript) is required for its association with the phosphopeptide-binding FHA domain in Cds1 (Kai et al., 2005). In turn, binding to Cds1 is a pre-requisite for Mus81 hyperphosphorylation, which induces its dissociation from chromatin without affecting nuclease activity (Figure 2B; Kai et al., 2005). Thus, the checkpoint-mediated modification of Mus81 is thought to prevent Mus81-dependent cleavage of replication/recombination intermediates generated after HU treatment (Kai et al., 2005).

Recent work has revealed a new layer of complexity in the regulation of Mus81-Eme1. Eme1 is phosphorylated in a Rad3 (ATR)- and Chk1 (CHK1)-dependent manner, both after treatment with genotoxic agents and in the absence of Rqh1 (Sgs1/BLM). Interestingly, the modification of Eme1 is Cds1-independent and causes a marked increase in the activity of Mus81 nuclease (Dehe et al., 2013). Moreover, Eme1 is also a substrate of the cyclin-dependent kinase Cdc2 (CDK), which modifies Eme1 in a cell cycle stage-specific manner (Dehe et al., 2013).

A comparative analysis of two Eme1 mutants, one refractory to phosphorylation events in response to camptothecin (CPT) treatment (Eme1^6SA^) and another carrying mutations in a subset of four Cdc2-consensus sites within Eme1^6SA^ (Eme1^4SA^; Figure 2B), revealed identical consequences to Mus81-Eme1 phospho-activation: neither Eme1^6SA^ nor Eme1^4SA^ could be phosphorylated or biochemically hyperactivated by CPT-treatment. Consequently, the authors have suggested that Cdc2-dependent phosphorylation is required as a priming event for the subsequent CPT-induced modification (Dehe et al., 2013).

Recent results have shown that both the intra-S and DNA-damage checkpoints are blind to the presence of the type of recombination intermediates that require Mus81-Eme1 for resolution at the onset of mitosis (Mohebi et al., 2015). Therefore, future analyses of Mus81-Eme1 regulation will be essential to unravel the intricate interconnections and relative contributions of the cell cycle and the checkpoint machineries for Mus81-Eme1 activation. This is particularly relevant given the stark contrast with the situation in budding yeast, where both physical and genetic evidence have ruled out that the DNA-damage checkpoint kinases like Mec1 (ATR) or Tel1 (ATM) contribute significantly to either the phosphorylation of the Mus81-Mms4 nuclease or the ensuing resolution of late recombination intermediates (Szakal and Branzei, 2013).

#### Human MUS81-EME1/EME2

Homology-based searches using *S. pombe* Eme1 as a bait revealed the existence of two partners for MUS81 in human cells, EME1 and EME2 (Figure 2C; Ciccia et al., 2003). While both complexes can process branched DNA structures *in vitro*, MUS81-EME2 exhibits higher nuclease activity and broader substrate specificity (Ciccia et al., 2007; Amangyeld et al., 2014; Pepe and West, 2014b). MUS81 and EME1 display increased levels of phosphorylation in prometaphase nocodazole-arrested cells compared to asynchronous, thymidine-(G1/S) or CPT-arrested (S/G2) cells. Given the coincidence of such modifications and an increase in the catalytic activity of MUS81 immunoprecipitates, it was put forward that similar regulatory mechanisms might operate to control MUS81 function in *S. cerevisiae* and in humans (Matos et al., 2011).

In terms of protein–protein interactions, MUS81 is also known to directly associate with SLX4. Together with SLX1, SLX4 constitutes a SSE with the ability to process recombination intermediates *in vitro* and *in vivo* and serves as a landing platform for other DNA repair factors like XPF-ERCC4 (Andersen et al., 2009; Fekairi et al., 2009; Munoz et al., 2009; Svendsen et al., 2009; Wechsler et al., 2011). The MUS81-SLX4 interaction is mediated through residues within the N-terminal region (1–86) of MUS81 and the SAP domain (a putative DNA-binding region) of SLX4 and plays and important role in both general HR repair as well as in CPT- and PARP inhibition-induced DNA damage repair (Fekairi et al., 2009; Castor et al., 2013; Garner et al., 2013; Kim et al., 2013). Mutations such as W24A/L25A and L66A/Q67A in the murine ortholog of MUS81 can disrupt the interaction with SLX4 without affecting the stability of the MUS81-EME1 complex or its nucleolytic activity on 3′-flaps (Nair et al., 2014). Likewise, the Y1340A, L1348A, and E1351A/L1352A mutations in the SLX4 SAP domain from mice could specifically abolish the MUS81-SLX4 interaction without disrupting the SLX4 ability to coimmunoprecipitate SLX1 and ERCC1 (Castor et al., 2013).

Interestingly, the increase in HJ-processing activity observed in MUS81 immunoprecipitates from cells arrested with nocodazole is dependent on CDK activity and requires SLX4 (Wyatt et al., 2013). SLX4, like MUS81 and EME1, is phosphorylated in a CDK1-dependent manner and inhibition of CDK kinase activity in nocodazole-arrested cells triggers dissociation of the complex. Therefore, it has been suggested that the increased capability of HJ resolution in mitotic MUS81 immunoprecipitates may arise from the coordination of different nucleases on the SLX4 scaffold (Wyatt et al., 2013), although the molecular basis for the CDK1-driven interaction of these two proteins remains unclear. In this sense, biochemical experiments have shown that full-length recombinant SLX1-SLX4 and MUS81-EME1 complexes can interact with each other *in vitro* to form a more efficient HJ resolvase. Quantitatively, the complex of the two nucleases displays higher HJ-processing activity than the sum of both nucleases separately, with a particular stimulation of the initial rate of the reaction. Qualitatively, the SLX1-SLX4-MUS81-EME1 complex carries out a more coordinated HJ resolution reaction, as judged by the increased rate of bilateral cleavage and linear product formation in a plasmid-borne cruciform cleavage assay (Wyatt et al., 2013). These results indicate that recruitment of the MUS81 nuclease to the SLX4 scaffold can improve its HJ resolution activity by coordinating its actions with those of SLX1.

Finally, another layer of complexity in the regulation of the MUS81 nuclease arises from the existence of the non-catalytic subunit EME2. While EME1 associates with MUS81 throughout the cell cycle (Matos et al., 2011; Wyatt et al., 2013), the MUS81-EME2 complex is detectable predominantly during S-phase (Pepe and West, 2014a). Therefore, the usage of alternative non-catalytic subunits may play a significant role in the regulation of MUS81, with MUS81-EME2 being involved in earlier events like replication fork restart, but not in later roles like the removal of HJs (Pepe and West, 2014a). We anticipate that forthcoming studies will refine our knowledge about the MUS81 partner choice (EME1 vs. EME2) and its connection to the distinct cellular functions of the two MUS81 complexes.

### The Yen1/GEN1 Nucleases

Human GEN1 and *S. cerevisiae* Yen1 are ortholog enzymes that belong to the subclass IV of the XPG/Rad2 family of SSEs (Johnson et al., 1998; Furukawa et al., 2003; Ip et al., 2008). Like all the other members of this family, they contain a bipartite nuclease domain, constituted by the XPG-N and XPG-I subdomains connected by a poorly conserved linker region (Lieber, 1997). While this is the main area for the interaction between the protein and the branched DNA region, another conserved feature of the family, the helix-two-turn-helix motif, stabilizes DNA binding through its contacts with the duplex DNA portion of the substrates (Tsutakawa et al., 2011, 2014). Yen1 and GEN1 were characterized as the first eukaryotic nucleases that processed HJs in a similar manner to the archetypical bacterial RuvC HJ resolvase (Ip et al., 2008). A recent report has shown that the two members of this subclass IV in *A. thaliana*, AtGEN1 and AtSEND1, also possess HJ resolution activity (Bauknecht and Kobbe, 2014), supporting the hypothesis that this subclass IV of the XPG/Rad2 family comprises a group of enzymes that have evolved HJ resolution activity (Ip et al., 2008). Interestingly, all these HJ resolvases retain the characteristic 5′-flap processing activity of the family, while they can also target other replication fork-like structures (Kanai et al., 2007; Ip et al., 2008; Rass et al., 2010; Yang et al., 2012; Bauknecht and Kobbe, 2014; Freeman et al., 2014). Therefore, as with MUS81, such potential for the cleavage of fully double-stranded replication or recombination intermediates could explain why cells have implemented mechanisms to tame these inherently dangerous activities.

#### *S. cerevisiae* Yen1

Two distinct layers of cell-cycle stage-specific regulation govern Yen1 function: subcellular localization and biochemical activation. The basis for this regulation relies on changes in the phosphorylation status of the protein, which are imposed by two master regulators of the cell cycle: Cdc28/Cdk kinase and Cdc14 phosphatase (Blanco et al., 2014; Eissler et al., 2014; Garcia-Luis et al., 2014). At the onset of S-phase, phosphorylation of Yen1 drives its exclusion from the nucleus, at the same time that it inhibits its nuclease activity. When cells enter anaphase, Cdc14 is released from the nucleolus and dephosphorylates Yen1, which re-enters the nucleus and becomes catalytically active (Kosugi et al., 2009; Matos et al., 2011, 2013; Blanco et al., 2014; Eissler et al., 2014; Garcia-Luis et al., 2014).

**FIGURE 3:**
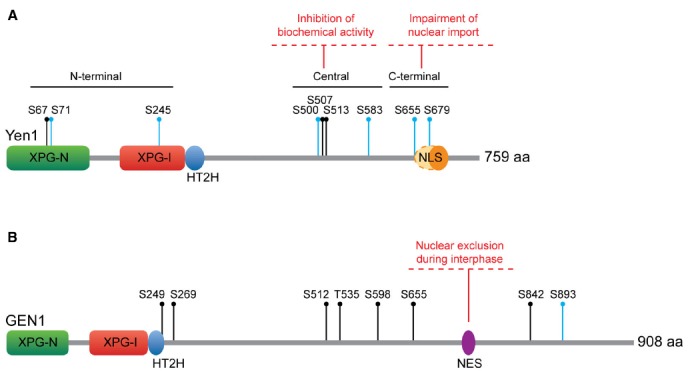
**Regulation of Yen1/GEN1. (A)** *S. cerevisiae* Yen1. Yen1 NLS has been traditionally considered monopartite (orange oval), although Eissler et al. (2014) have recently proposed it could be bipartite (extending to the dashed oval area). All the Cdk sites are indicated. **(B)** Human GEN1. All the CDK sites are indicated. Key as in Figure 1.

Yen1 contains nine consensus Cdk sites (S-P, all serines), with eight of them being full Cdk sites S-P-X-K/R. These Cdk sites show some degree of clustering in each of the N-terminal, central and C-terminal regions of the protein (Figure 3A), a predictive feature of bona fide Cdk substrates (Moses et al., 2007). Indeed, Yen1 was confirmed as a target for Cdk-dependent phosphorylation in whole-cell extracts through proteome-wide approaches (Ubersax et al., 2003), being a particularly good substrate for the S-phase complex Cdc28-Clb5 (Loog and Morgan, 2005). Six out of these nine Cdk sites have been verified as phosphoresidues *in vivo* by mass spectrometry (Blanco et al., 2014; Eissler et al., 2014). Additionally, four of the Cdk sites were identified as optimal targets for Cdc14 (S500, S507, S655, and S679) through *in silico* prediction and *in vitro* analyses of Cdc14 activity on peptides containing these phosphoresidues (Figure 3A; Eissler et al., 2014).

With regard to the control of Yen1 nuclease activity, it was initially observed that the ability of Yen1 to process a HJ substrate fluctuates throughout the cell cycle. While immunoprecipitates of Yen1 from cells in S-phase show low levels of nuclease activity, those from cells in mitosis can efficiently process synthetic HJs (Matos et al., 2011). Mutation of the nine CDK consensus sites in Yen1 to alanine results in a protein [Yen1-9A (Eissler et al., 2014) or Yen1^ON^ (Blanco et al., 2014)] that, as opposed to the wild-type, (i) cannot be phosphorylated by Cdk, (ii) does not interact with Cdc14 and (iii) displays a maximum level of activity during all phases of the cell cycle, bypassing the requirement for Cdc14 for its activation. A partial dissection of the relative contribution of the different Cdk sites to this regulation has shown that serine to alanine mutations in the C-terminal cluster (S655 and S679) have no effect on the biochemical activity of Yen1 and, so far, the significance of the potential phosphorylation events on the N-terminal cluster remains poorly defined. However, phosphorylation-resistant mutants in the four serines of the central cluster (S500, S507, S513, and S583) result in a protein that is no longer inhibited during S-phase and displays increased levels of crossover formation (Blanco et al., 2014; Eissler et al., 2014). Conversely, expression of a phosphomimetic mutant for these residues phenocopies the deletion of *YEN1* (Eissler et al., 2014). Since phosphorylation-dependent inhibition of Yen1 nuclease activity derives from a reduction in its substrate binding affinity (Blanco et al., 2014), the central cluster may be part of a repressible DNA binding domain or serve as a switch for a conformational change between low- and high-DNA binding forms of Yen1.

Concerning the spatial regulation of Yen1, the changes in its localization are related to the phosphorylation status of the CDK target site S679. This serine in the C-terminal cluster overlaps with a predicted nuclear localization signal (NLS; 679-SPIKKSRTT-687). Immunofluorescence analysis of overexpressed, GFP-tagged Yen1 revealed that the mutation of S679 to alanine led to permanent nuclear accumulation (Kosugi et al., 2009). A more detailed analysis showed that phosphorylation of other CDK sites may also influence Yen1 localization. The proportion of nuclear Yen1 was shown to be higher with Yen1^ON^ than with Yen1*^S679A^* mutants (Blanco et al., 2014). Therefore, it has been suggested that the NLS in Yen1 may be bipartite and controlled by the phosphorylation of two different CDK sites, S655, and S679 (Eissler et al., 2014). In addition, the observation that Msn5, a karyopherin involved in the nuclear export of phosphorylated proteins, is responsible for Yen1 export (Kosugi et al., 2009), suggests that the phosphorylation status of CDK sites overlapping yet-unidentified nuclear export signals may also influence its localization.

The expression of Yen1^ON^/Yen1-9A, which bypasses the two levels of Cdk-dependent control, has demonstrated the importance of restricting Yen1 function prior to anaphase for the maintenance of genome stability. Premature activation of Yen1 leads to increased DNA-damage sensitivity, crossover formation and loss of heterozygosity in diploid cells (Blanco et al., 2014; Eissler et al., 2014). Incidentally, Yen1^ON^ can suppress the synthetic lethality of *mus81*Δ *sgs1*Δ double mutants, thus demonstrating that the premature activation of Yen1 can compensate for the loss of other genes involved in the processing of recombination intermediates (Blanco et al., 2014).

#### Human GEN1

Soon after its identification, several lines of evidence pointed toward proteolytic cleavage as a putative mechanism to regulate GEN1 activity through the excision of a self-inhibitory domain in the long, unstructured C-terminal region of the protein (Ip et al., 2008; Rass et al., 2010). Both the ∼60 kD N-terminal fragment originally identified by mass spectrometry of highly fractionated HeLa extracts and the recombinant GEN1^1–527^ truncation fragment exhibited increased biochemical activity compared to the full-length protein (Ip et al., 2008). Moreover, GEN1^1–527^ was able to partially suppress the DNA damage sensitivity and meiotic crossover defects of fission yeast strains deficient for either Mus81 or Rqh1 (Sgs1 in budding yeast, BLM in mammalians; Lorenz et al., 2010). However, there is no definite evidence so far for the C-terminal region cleavage as an activation mechanism for GEN1 *in vivo*.

On the other hand, the sequence of GEN1 contains eight CDK consensus target sites, a number similar to those in Yen1, although their relative position and context is not conserved (Figure 3B). This suggested that a similar CDK phosphorylation-dependent regulatory mechanism could operate for Yen1 and GEN1 (Matos et al., 2011). It has been recently shown that GEN1 is indeed phosphorylated in a seemingly CDK-dependent manner, as mutation of all the serines/threonines in its consensus CDK targets (GEN1^8A^) abolishes virtually all the slowly migrating forms of the protein (Chan and West, 2014). However, changes in the phosphorylation status of the protein do not affect its nuclease activity as dramatically as in the case of Yen1, since both GEN1^8A^ and *in vitro* dephosphorylated GEN1 retain the wild-type ability to process HJs (Matos et al., 2011; Chan and West, 2014). Therefore, it appears unlikely that the control of the biological functions of GEN1 relies on the direct modulation of its nucleolytic activity. Instead, localization studies have suggested that human cells restrict the actions of this nuclease through its temporal exclusion from the nucleus. GEN1 is strongly enriched in the cytoplasm during interphase, gaining access to chromatin in prometaphase, only after the nuclear envelope has broken down (Matos et al., 2011). The subcellular localization pattern of GEN1 appears to be exclusively dependent on a nuclear export signal that has been recently identified in its unstructured C-terminal region (660-LLSGITDLCL-669; Chan and West, 2014).

To demonstrate the importance of the restriction of GEN1 access to the nucleus prior to mitosis, a constitutively nuclear version of the enzyme, GEN1^nuc^, was generated by adding three copies of a SV40-derived NLS at its C-terminus and by mutating four key hydrophobic residues in GEN1 NES to alanine (L660A, L661A, I664A, L667A). When introduced in cells, GEN1^nuc^ induces a series of phenotypes that are partially reminiscent of those observed in yeast expressing mis-regulated Yen1. For instance, GEN1nuc expression increases the occurrence of sister chromatid exchanges (COs). Also, it can reduce the defects associated with the double depletion of MUS81 and BLM, resulting in increased cellular viability and reduction of chromosomal breaks (Chan and West, 2014). However, no increased sensitivity to DNA-damaging agents was observed in cells expressing GEN1^nuc^. This could reflect a differential ability between Yen1 and GEN1 to process branched DNA structures that are generated *in vivo* during active replication, stalled replication fork repair/restart or the early steps of homologous recombination (Blanco et al., 2014; Chan and West, 2014).

## Concluding Remarks

Traditional models of homologous recombination based on a DNA substrate-centric view of JM-processing (e.g., Figure 1) are insufficient to explain how cells control the outcome of DNA repair. In such models, enzymes are positioned according to their *in vitro* substrate preference, which is difficult to reconcile with their rather promiscuous biochemical properties and intricate genetic relationships. Work on the regulation of MUS81* and Yen1/GEN1 nucleases has introduced a new dimension to such models, a dimension in which defined cellular circumstances strongly influence pathway usage. However, despite the recent advances summarized here, there are still fundamental questions concerning SSE regulation that remain unanswered. For instance, we have just begun to comprehend the mechanistic basis for the control of the catalytic properties of SSEs by post-translational modifications. Therefore, we will need detailed biochemical and structural information to help us understand how similar phosphorylation events translate into opposite responses from each protein.

The ability to turn on and off JM-processing enzymes at a given cell cycle stage, or upon the cellular detection of a given stimulus, seems an efficient mechanism to bias the choice toward the most suitable DNA repair pathway and could potentially control the function of repair enzymes other than MUS81* and Yen1/GEN1. It is therefore tempting to envisage that in addition to developing enzymes capable of processing a specialized, but overlapping range of DNA substrates, cells have evolved the general ability to regulate their actions. Such combination of capacities would prevent pathway competition as well as the toxic processing of vital endogenous DNA structures. Furthermore, it would allow for the flexible implementation of pathway usage according to the chromosome segregation programs of meiosis and mitosis.

### Conflict of Interest Statement

The authors declare that the research was conducted in the absence of any commercial or financial relationships that could be construed as a potential conflict of interest.
